# Supplementary material for: Children’s Intake of Food from Non-Fast-Food Outlets and Child-Specific Menus: A Survey of Parents
Source: Children (Basel). 2019 Nov 1;6(11):123. doi: 10.3390/children6110123 (PMC6915613; doi:10.3390/children6110123)
Supplement: Supplementary file 1 [file children-06-00123-s001.pdf]

Table S1. Survey responses (*n* (%)) stratified by socio-demographic characteristics.

	**Total Sample**	**Child Sex**		**Parent Sex**		**Parental Education**			**Household Annual Income (AUD)**				**Number of Children**					
		Girl	Boy	Female	Male	HSC	TAFE/Diploma	Tertiary institute	$20,000-$40,000	$40,000-$60,000	$60,000-$80,000	>$80,000	1	2	3	4	5	6
Q1 [a]																		
Never	21 (22)	10 (23)	10 (20)	19 (24)	2 (12)	1 (13)	6 (21)	14 (24)	0 (0)	0 (0)	0 (0)	20 (27)	4 (33)	13 (22)	3 (15)	0 (0)	1 (100)	0 (0)
< Once a month	21 (22)	10 (23)	11 (22)	16 (21)	5 (29)	1 (13)	7 (25)	13 (22)	0 (0)	3 (75)	2 (22)	15 (20)	2 (17)	14 (24)	5 (25)	0 (0)	0 (0)	0 (0)
< Once a week	21 (22)	9 (20)	12 (24)	18 (23)	3 (18)	2 (25)	5 (18)	14 (24)	1 (33)	0 (0)	3 (33)	17 (23)	2 (17)	12 (21)	5 (25)	2 (67)	0 (0)	0 (0)
1–2 times weekly	13 (14)	7 (16)	5 (10)	8 (10)	5 (29)	2 (25)	5 (18)	6 (10)	0 (0)	0 (0)	4 (44)	8 (11)	2 (17)	9 (16)	1 (5)	0 (0)	0 (0)	1 (100)
3–4 times weekly	10 (11)	4 (9)	6 (12)	8 (10)	2 (12)	1 (13)	1 (4)	8 (14)	0 (0)	1 (25)	0 (0)	8 (11)	0 (0)	7 (12)	2 (10)	1 (33)	0 (0)	0 (0)
5–6 times weekly	9 (9)	4 (9)	5 (10)	9 (12)	0 (0)	1 (13)	4 (14)	4 (7)	2 (67)	0 (0)	0 (0)	7 (9)	2 (17)	3 (5)	4 (20)	0 (0)	0 (0)	0 (0)
Total	95 (100)	44 (100)	49 (100)	78 (100)	17 (100)	8 (100)	28 (100)	59 (100)	3 (100)	4 (100)	9 (100)	75 (100)	12 (100)	58 (100)	20 (100)	3 (100)	1 (100)	1 (100)
Q2 [a]																		
Never	8 (8)	6 (14)	2 (4)	7 (9)	1 (6)	1 (13)	4 (14)	3 (5)	1 (33)	0 (0)	2 (22)	5 (7)	0 (0)	6 (10)	2 (10)	0 (0)	0 (0)	0 (0)
< Once a month	36 (38)	18 (41)	16 (33)	28 (36)	8 (47)	4 (50)	9 (32)	23 (39)	0 (0)	1 (25)	1 (11)	33 (44)	7 (58)	22 (38)	4 (20)	1 (33)	1 (100)	1 (100)
< Once a week	34 (36)	12 (27)	22 (45)	29 (37)	5 (29)	2 (25)	10 (36)	22 (37)	0 (0)	3 (75)	4 (44)	25 (33)	4 (33)	21 (36)	8 (40)	1 (33)	0 (0)	0 (0)
1–2 times weekly	16 (17)	8 (18)	8 (16)	13 (17)	3 (18)	1 (13)	5 (18)	10 (17)	2 (67)	0 (0)	2 (22)	11 (15)	1 (8)	8 (14)	6 (30)	1 (33)	0 (0)	0 (0)

	Total Sample	Child Sex		Parent Sex		Parental Education			Household Annual Income (AUD)				Number of Children					
		Girl	Boy	Female	Male	HSC	TAFE/Diploma	Tertiary institute	$20,000-$40,000	$40,000-$60,000	$60,000-$80,000	>$80,000	1	2	3	4	5	6
3–4 times weekly	1 (1)	0 (0)	1 (2)	1 (1)	0 (0)	0 (0)	0 (0)	1 (2)	0 (0)	0 (0)	0 (0)	1 (1)	0 (0)	1 (2)	0 (0)	0 (0)	0 (0)	0 (0)
Total	95 (100)	44 (100)	49 (100)	78 (100)	17 (100)	8 (100)	28 (100)	59 (100)	3 (100)	4 (100)	9 (100)	75 (100)	12 (100)	58 (100)	20 (100)	3 (100)	1 (100)	1 (100)
Q3																		
Often	23 (26)	10 (26)	13 (28)	20 (28)	3 (19)	4 (57)	6 (25)	13 (23)	0 (0)	1 (25)	2 (29)	19 (27)	4 (33)	12 (23)	5 (28)	0 (0)	1 (100)	1 (100)
Sometimes	24 (28)	11 (29)	13 (28)	19 (27)	5 (31)	2 (29)	7 (29)	15 (27)	1 (50)	2 (50)	1 (14)	19 (27)	3 (25)	17 (33)	3 (17)	1 (33)	0 (0)	0 (0)
Rarely	29 (33)	12 (32)	15 (32)	23 (32)	6 (38)	0 (0)	9 (38)	20 (36)	1 (50)	1 (25)	3 (43)	23 (33)	3 (25)	18 (35)	7 (39)	1 (33)	0 (0)	0 (0)
Never	8 (9)	3 (8)	5 (11)	7 (10)	1 (6)	1 (14)	1 (4)	6 (11)	0 (0)	0 (0)	0 (0)	7 (10)	2 (17)	2 (4)	3 (17)	1 (33)	0 (0)	0 (0)
I don't know/can't say	3 (3)	2 (5)	1 (2)	2 (3)	1 (6)	0 (0)	1 (4)	2 (4)	0 (0)	0 (0)	1 (14)	2 (3)	0 (0)	3 (6)	0 (0)	0 (0)	0 (0)	0 (0)
Total	87 (100)	38 (100)	47 (100)	71 (100)	16 (100)	7 (100)	24 (100)	56 (100)	2 (100)	4 (100)	7 (100)	70 (100)	12 (100)	52 (100)	18 (100)	3 (100)	1 (100)	1 (100)
Q4																		
Every time	5 (7)	2 (6)	3 (7)	5 (8)	0 (0)	1 (17)	1 (5)	3 (6)	0 (0)	0 (0)	0 (0)	5 (8)	0 (0)	4 (9)	1 (7)	0 (0)	0 (0)	0 (0)
Often	22 (29)	9 (27)	11 (27)	20 (32)	2 (14)	2 (33)	8 (36)	12 (25)	1 (50)	2 (50)	1 (17)	17 (28)	1 (10)	16 (34)	4 (27)	0 (0)	1 (100)	0 (0)
Sometimes	32 (42)	12 (36)	20 (49)	24 (39)	8 (57)	3 (50)	8 (36)	21 (44)	1 (50)	1 (25)	2 (33)	26 (43)	7 (70)	16 (34)	7 (47)	1 (50)	0 (0)	1 (100)
Rarely	12 (16)	7 (21)	5 (12)	10 (16)	2 (14)	0 (0)	4 (18)	8 (17)	0 (0)	1 (25)	2 (33)	9 (15)	1 (10)	8 (17)	2 (13)	1 (50)	0 (0)	0 (0)

	Total Sample	Child Sex		Parent Sex		Parental Education			Household Annual Income (AUD)				Number of Children					
		Girl	Boy	Female	Male	HSC	TAFE/Diploma	Tertiary institute	$20,000-$40,000	$40,000-$60,000	$60,000-$80,000	>$80,000	1	2	3	4	5	6
Never	5 (7)	3 (9)	2 (5)	3 (5)	2 (14)	0 (0)	1 (5)	4 (8)	0 (0)	0 (0)	1 (17)	4 (7)	1 (10)	3 (6)	1 (7)	0 (0)	0 (0)	0 (0)
Total	76 (100)	33 (100)	41 (100)	62 (100)	14 (100)	6 (100)	22 (100)	48 (100)	2 (100)	4 (100)	6 (100)	61 (100)	10 (100)	47 (100)	15 (100)	2 (100)	1 (100)	1 (100)
Q5																		
Yes	65 (86)	28 (85)	35 (85)	54 (87)	11 (79)	5 (83)	21 (95)	39 (81)	2 (100)	4 (100)	6 (100)	51 (84)	8 (80)	41 (87)	12 (80)	2 (100)	1 (100)	1 (100)
No	8 (11)	4 (12)	4 (10)	5 (8)	3 (21)	0 (0)	1 (5)	7 (15)	0 (0)	0 (0)	0 (0)	7 (11)	1 (10)	4 (9)	3 (20)	0 (0)	0 (0)	0 (0)
I don't know/can't say	3 (4)	1 (3)	2 (5)	3 (5)	0 (0)	1 (17)	0 (0)	2 (4)	0 (0)	0 (0)	0 (0)	3 (5)	1 (10)	2 (4)	0 (0)	0 (0)	0 (0)	0 (0)
Total	76 (100)	33 (100)	41 (100)	62 (100)	14 (100)	6 (100)	22 (100)	48 (100)	2 (100)	4 (100)	6 (100)	61 (100)	10 (100)	47 (100)	15 (100)	2 (100)	1 (100)	1 (100)
Q6																		
Too large	26 (34)	7 (21)	19 (46)	22 (35)	4 (29)	2 (33)	7 (32)	17 (35)	1 (50)	2 (50)	2 (33)	20 (33)	2 (20)	14 (30)	7 (47)	1 (50)	1 (100)	1 (100)
Just right	29 (38)	16 (48)	13 (32)	25 (40)	4 (29)	3 (50)	9 (41)	17 (35)	1 (50)	2 (50)	2 (33)	22 (36)	3 (30)	19 (40)	6 (40)	1 (50)	0 (0)	0 (0)
Too small	16 (21)	7 (21)	7 (17)	12 (19)	4 (29)	1 (17)	5 (23)	10 (21)	0 (0)	0 (0)	1 (17)	15 (25)	4 (40)	11 (23)	1 (7)	0 (0)	0 (0)	0 (0)
Don't know	5 (7)	3 (9)	2 (5)	3 (5)	2 (14)	0 (0)	1 (5)	4 (8)	0 (0)	0 (0)	1 (17)	4 (7)	1 (10)	3 (6)	1 (7)	0 (0)	0 (0)	0 (0)
Total	76 (100)	33 (100)	41 (100)	62 (100)	14 (100)	6 (100)	22 (100)	48 (100)	2 (100)	4 (100)	6 (100)	61 (100)	10 (100)	47 (100)	15 (100)	2 (100)	1 (100)	1 (100)

Q: Question; HSC: Higher School Certificate; TAFE: Technical and Further Education. Results are presented in *n* (%), the total may not sum up to 100% due to missing responses in incomplete surveys. [a] For Q1 and Q2, "every day" was given as a response option but none of the participants selected this. The survey

questions were: Q1. How often does your child/ren have meals or snacks such as burgers, pizza, chicken, or chips from places like McDonalds, Hungry Jacks, Pizza Hut, KFC, Red Rooster or local takeaway food places? (include eat-in, takeaway or home delivered foods). Q2. How often does your child/ren have meals or snacks from a bistro, café or non-fast food restaurant (include eat-in, takeaway or home delivered foods). Q3. When you have a meal or snack, with your child/ren, from a bistro, café or non-fast food restaurant, how often do you see that a "children's' menu" or a "kids' menu" is available? Q4. When ordering a meal or snack for your child/ren (6 months to 12 years of age) from a bistro, café or non-fast food restaurant, have you or your child/ren ever ordered from a "children's' menu" or a "kids' menu"? Q5. Are there any changes that you would like to see made to "children's' menus" or "kids' menus" at bistros, cafés or non-fast food restaurants? Q6. At a typical bistro, café or non-fast food restaurant, how would you describe the overall portion size of options on "kid's menus" or "kids' menus"?
